# Development, stabilization, and characterization of nanoemulsion of vitamin D_3_-enriched canola oil

**DOI:** 10.3389/fnut.2023.1205200

**Published:** 2023-08-24

**Authors:** Aafia Khalid, Muhammad Umair Arshad, Ali Imran, Syed Haroon Khalid, Mohd Asif Shah

**Affiliations:** ^1^Department of Food Science, Government College University, Faisalabad, Pakistan; ^2^Department of Pharmaceutics, Government College University, Faisalabad, Pakistan; ^3^School of Business, Woxsen University, Hyderabad, Telangana, India; ^4^Division of Research and Development, Lovely Professional University, Phagwara, Punjab, India; ^5^School of Engineering and Technology, Sharda University, Greater Noida, India; ^6^Department of Economics, Kabridahar University, Somali, Ethiopia

**Keywords:** nanoemulsion, canola oil, water titration techniques, vitamin D fortification, bioavailability

## Abstract

In this study, the oil-in-water nanoemulsion (NE) was prepared and loaded with vitamin D_3_ in food-grade (edible) canola oil and stabilized by Tween 80 and Span 80 by using a water titration technique with droplet sizes of 20 to 200 nm. A phase diagram was established for the influence of water, oil, and S-Mix concentration. The outcomes revealed that the particle size of blank canola oil nanoemulsion (NE) ranged from 60.12 to 62.27 (d.nm) and vitamin D_3_ NE ranged from 93.92 to 185.5 (d.nm). Droplet size and polydispersity index (PDI) of both blank and vitamin D_3_-loaded NE results were less than 1, and zeta potential results for blank and vitamin D_3_ loaded NE ranged from −9.71 to −15.32 mV and −7.29 to −13.56 mV, respectively. Furthermore, the pH and electrical conductivity of blank NE were 6.0 to 6.2 and 20 to 100 (μs/cm), respectively, whereas vitamin D_3_-loaded NE results were 6.0 to 6.2 and 30 to 100 (μs/cm), respectively. The viscosity results of blank NE ranged from 0.544 to 0.789 (mPa.s), while that of vitamin D_3_-loaded NE ranged from 0.613 to 0.793 (mPa.s). In this study, the long-term stability (3 months) of canola oil NE containing vitamin D3 at room temperature (25 C) and high temperature (40 C) was observed.

## 1. Introduction

Due to their effectiveness and efficacy, nanoemulsion has an eminent place among the many drug delivery strategies. During their formulation, lipophilic active compounds including nutraceuticals, drugs, and flavors are incorporated into the aqueous medium of pharmaceuticals and food products. The emulsion is defined as the merging of two immiscible liquids together in the presence of an emulsifying agent, in which one liquid is dispersed into another, referred to as the dispersed phase and the continuous phase (1, 2). Moreover, colloidal suspensions with very minute droplets, generally 20–200 nm in diameter, distributed in a liquid medium are known as nanoemulsions (NEs) (3). Due to the changes in physiochemical properties and biological performances related to the alteration in particle size, nanoemulsions are extensively utilized in the food industry. These nanodroplets in emulsion-based delivery systems are beneficial in the field of the food industry due to their higher optical clarity, increased oral bioavailability, and greater stability for droplet aggregation separation (4, 5). For the administration of probiotics, nutraceuticals, flavors, and colors, nanoemulsions hold interesting potential and have been shown to be one of the most effective methods for protecting sensitive compounds' characteristics (6, 7). These are kinetically stable and thermodynamically unstable; however, their physical stability without coalescence makes them distinctive conventional emulsions (8). The majority of nutrients are passed through the digestive process in an oil-in-water emulsion, which can be categorized as oil-in-water in which oil droplets are dragged in a continuous water phase or water-in-oil in which water droplets are submerged in a continuous oil phase (9). The size of all the particles may tend toward nano to mixed micelle array before the time of absorption. However, the majority of people are deficient in vitamin D, which may lead to rickets, osteomalacia, and bone disease. Due to its lower stability in the food chain, the beverage industry's fortification of food products with fat-soluble vitamins has proved problematic (10, 11). For oral consumption, emulsion-based delivery of fat-soluble vitamin D3 can be a suitable method (12). The previous studies demonstrated the formation of nano-phytosomes using lipid- and solvent-based derivative methods (13), micro/nanoemulsion formulations (14), encapsulation of vitamin D_3_, loaded with cyclodextrin (15), zein nanodroplets enveloped with carboxymethyl chitosan (12), phase inversion temperature method (16) and preparation, modeling characterization, and release profile of vitamin D3 nanoemulsion (17). Therefore, using nanotechnology to encapsulate vitamin D into nano-delivery systems and then using them for additional fortification is an efficient way to increase solubility, bioavailability, distribution, and protection against oxidation, UV light, and manufacturing conditions (16). Canola oil has health-supporting nutritional properties due to its good source of oleic acid (50–60% portion), α-linolenic acid (6–14% portion), and some unsaturated fatty acids (18). Moreover, canola oil exhibited strong anti-oxidant properties due to the presence of phenolic compounds, primarily sinapic acid and its derivatives (19). In many studies, these two surfactants (Tween 80 and Span 80) show high compatibility to build a stable interfacial film and give better performance during emulsification process studies (20–22). Canola oil, distilled water, the surfactant Tween 80, and the co-surfactant Span 80 were specifically generated in this study's canola oil-enhanced vitamin D_3_ oil-in-water nanoemulsion formulations utilizing the water titration technique. The physicochemical characteristics of nanoemulsions were examined by a pseudo-ternary phase diagram, electrical conductivity, pH, viscosity, droplet size distribution, thermal stability, and shelf life study.

## 2. Materials and methods

### 2.1. Materials

Pure canola oil was purchased from the local market in Faisalabad (Pakistan). Span 80 and Tween 80 were purchased from Daejung. Distilled water was used in emulsions and solutions. Vitamin D_3_ (cholecalciferol) was kindly gifted by Textile Testing Laboratories in Lahore. All chemical reagents were used for analytical and food grades.

### 2.2. Construction of the Pseudo-Ternary phase diagram

Canola oil, surfactant (Tween 80), and co-surfactant (Span 80) (S/Co-S) were used for nanoemulsion formulation. For the production of the pseudo-ternary phase diagram, the surfactant/co-surfactant (S/Co-s) ratio of 1:1 was adopted. The water titration technique was used to create a pseudo-ternary phase structure to locate the nanoemulsion zone and determine the concentration ratio of the constituent ingredients (23). During the experimental phase, in different falcon tubes, oil was thoroughly mixed with surfactant/co-surfactant (S/Co-s) by using a 2-min vortex mixer (PCSIR, Pakistan) at different weight ratios of 0.95:0.05, 0.9:0.1, 0.85:0.15, 0.8: 0.20, 0.75:0.25, 0.70:0.3, 0.65:0.35, 0.6:0.4, 0.55:0.45, 0.5:0.5, 0.4:0.6, 0.3:0.7, 0.2:0.8, 0.1:0.9, and 0.05:0.95 (w/w). The aqueous phase was then added with an increment of 50 μl into each tube (at 26 ± 2°C) followed by 2 min of mixing. After homogenization, samples were visually observed by enlightening the sample with white light to confirm the nature of the sample. The resultant mixture is described as transparent, turbid, gel-like, or milky in physical appearance. The milky emulsion was declared a nanoemulsion (24). Chemix (cxse 700) was utilized to construct the ternary diagram.

### 2.3. Preparation of nanoemulsion formulae

The pseudo-ternary phase diagram's appropriate weight proportions for S/Co-S and oil were chosen, and several equations were created based on the diagram's nanoemulsion (NE) area. Due to the much smaller oil-to-water weight ratio, nanoemulsions were anticipated to be made with these weight proportions. In these NE systems, the physical condition and milkiness were seen.

### 2.4. Preparation of vitamin D_3_-loaded nanoemulsion formulae

The nanoemulsions were prepared after considering the daily suggested intake of vitamin D (600 IUs). Vitamin D_3_ (Cholecalciferol) was added to the oil, and after 2 min of vigorous vortex mixing, a 1:1 combination of Tween 80 and Span 80 was added, and further mixing was performed continuously. Finally, to create a homogenous nanoemulsion combination, a specific weight of liquid phases was incorporated drop by drop (25).

### 2.5. Droplet size and polydispersity index

Dynamic light scattering was used to measure the droplet size and polydispersity index of a vitamin D3 nanoemulsion at a temperature of 25°C (Malvern Zeta Sizer Ver. 7.11, Malvern Instruments, UK). The word “droplet size” refers to nm. The outcomes of each measurement were determined using the mean and standard error. After the proper dilution, test samples were placed into sample cells. To ensure that the nanoemulsions' aggregate diameter was uniform, polydispersity index values were applied (26).

### 2.6. Zeta potential

By applying an electric field of one volt, the Malvern Zetasizer Nano ZS (Malvern Instruments, Ltd., UK) Ver. 7.11 with electrophoretic light scattering and a particulate size detector was used to evaluate the zeta potential of NE formulas. The samples were adequately diluted with pre-filtered double-distilled water for the zeta potential measurement, and measurements were made in triplicate (27).

### 2.7. pH and conductivity

pH and conductivities of formulated nanoemulsion were determined by using a portable pH/EC temperature meter (HANNA Instruments HI9811-5, Romania) as per the guidelines of (28).

### 2.8. Viscosity

Viscosity was determined by using a viscometer (BROOKFIELD Instruments, DV2TRVTJO, USA). To assure accuracy, measurements were performed three times, and average results were noted (29). Using a viscometer, a two-number spindle was coated with nanoemulsion and revolved at speeds of 5, 10, 20, and 50 rpm at 25°C (30) and a shear rate range of 3 and 218/s with some modifications. Readings on the viscometer were recorded for each speed. Mean values were computed when the samples were repeated in triplicate. The dispersion medium was purified water (20 ml), and the nanoemulsion (2 g) was weighed. For pH meter calibration, buffer solutions with pH values of 4, 7, and 9 were utilized. Mean values were calculated after the samples were repeated in triplicate (31).

### 2.9. Stability studies

Stability analyses of nanoemulsions containing vitamin D_3_ were evaluated. Each formulation of 5 ml was added into glass vials, sealed, and kept at room temperature (25 ± 0.5 C) and 40 C for 3 months. Then, samples were visually analyzed for milkiness, flocculation, phase separation, zeta potential, droplet size, PDI viscosity, pH, and conductivity. All studies were conducted in triplicate (32).

### 2.10. Statistical analysis

For data analysis, SPSS version 20 was utilized, and all findings were presented as mean and standard deviation (SD).

## 3. Results and discussion

### 3.1. Development of a pseudo-ternary diagram

**Figure 1:**
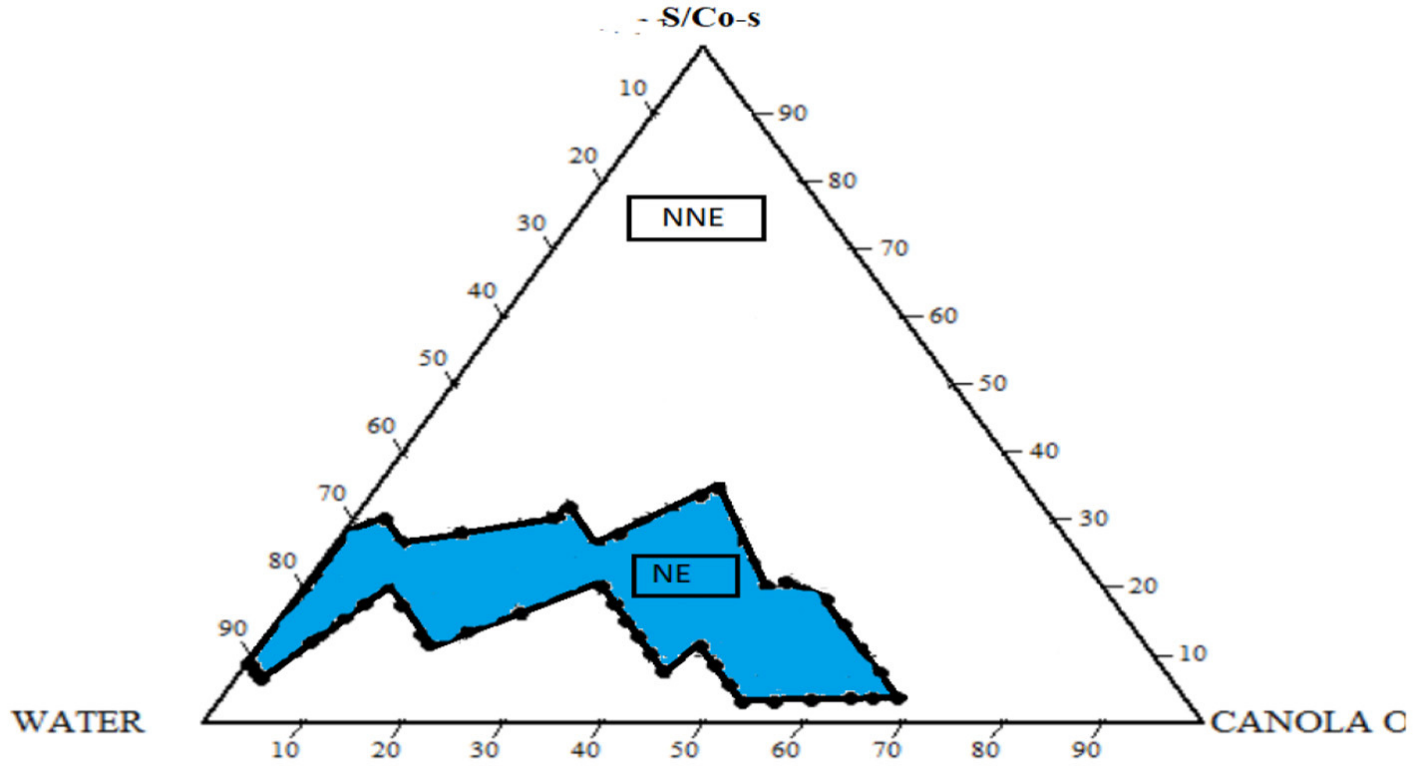
Pseudo-ternary phase diagram consisting of water, canola oil, and S/Co-s (Tween 80 and Span 80).

The isolation of the nanoemulsion zone devoid of vitamin D3 and the optimization of concentration for oil, surfactant, and co-surfactant were both designed using a pseudo-ternary phase diagram. For the ternary diagram's creation, a ratio of 1:1 was used. The colored region in Figure 1 denotes the nanoemulsion zone.

### 3.2. Selection of formulae

Even though several formulations could be chosen from the secured nanoemulsion region but only four formulae were selected to develop nanoemulsion, as shown in Table 1. It is documentary supported that the composition of a nanoemulsion may affect many characteristics of nanoemulsion; so, the oil surfactant and co-surfactant (S-Mix) in different concentrations were taken at 5% intervals.

**Table 1:** Optimum formulae for nanoemulsion preparation.

	**Composition (% w/w)**		
**Formulation code**	**Oil**	**S-Mix**	**Water**
CA-1	5%	30%	65%
CA-2	10%	35%	55%
CA-3	15%	40%	45%
CA-4	20%	40%	40%

### 3.3. Characterization and evaluation of vitamin D_3_-loaded formulations

#### 3.3.1. Visual inspection

All the formulations (blank and vitamin D_3_-loaded) were physically fairly stable and did not indicate precipitation, phase separation, and color change.

#### 3.3.2. Droplet size and PDI

Nanoemulsion size decreased to < 200 nm (33) as a result of vitamin D_3_-loaded formulations showing that the droplet size was reduced by increasing the S-Mix ratio (34). The small particle size was found to prevent flocculation, facilitating the system to remain dispersed with no phase separation. These findings imply that monounsaturated-rich oils, such as maize oil, are superior to polyunsaturated-rich oils, such as flaxseed or fish oil, at encapsulation and delivering the D3 vitamin. The conclusions revealed here may help in the development of more effective plant-derived vitamin-fortified meals and drinks reported by Schoener et al. (35). Similar results were shown by Maurya and Aggarwal (36), and the chosen nanodroplet size, zeta potential, and the durability of vitamin D3 storage under various environmental stress conditions were also examined. (i) moisture and the temperature: among them are (a) the accelerated condition (45 ± 2°C and RH 75 ± 5%), (b) the ambient condition (25 ± 3°C and RH 65 ± 5%), and (c) the chilled condition (six two °C and RH 55 ± 5%); (ii) pH (3–7) in a chilled environment; and (iii) ionic strength: NaCl concentration (0, 250, 500, and 750 mM) in a chilled environment. The values of droplet size of blank nanoemulsion formulations were found to range from 60.12 to 66.48 nm, and nanoemulsions with vitamin D_3_-loaded ranged from 93.92 to 185.5 nm as shown in Table 2. The PDI results show that the droplets are distributed uniformly throughout the compositions. The PDI value for each formulation is < 1.0. The value of PDI of blank formulations was found to range from 0.36 to 0.50, and PDI values of vitamin D_3_-loaded formulations were found to range from 0.29 to 0.93. All values of the nanoemulsion formulations were observed to be < 1 in this investigation.

**Table 2:** Droplet size and PDI of blank and vitamin D_3_-loaded nanoemulsion (mean ± SD *n* = 3).

	**Droplet size (d.nm)**		**PDI**	
**Formulation code**	**Blank**	**Vitamin D_3_ loaded**	**Blank**	**Vitamin D_3_-loaded**
CA-1	60.12 ± 2.46^b^	93.92 ± 2.44^d^	0.366^c^	0.293^d^
CA-2	61.78 ± 1.66^b^	113.6 ± 0.81^c^	0.384^bc^	0.440^c^
CA-3	62.27 ± 1.63^ab^	158.4 ± 2.44^b^	0.439^b^	0.755^b^
CA-4	66.48 ± 0.88^a^	185.5 ± 4.08^a^	0.508^a^	0.939^a^

Values are expressed as mean ± standard deviation (*n* = 3). Values in the same column within each parameter with different letters were significantly different from each other (*p* ≤ 0.05).

#### 3.3.3. Zeta potential

Hippalgaonkar et al. (37) stated that the zeta potential is the potential difference between the stationary layer and the dispersion medium of a fluid. Zeta potential is an electro-kinetic potential, and nanoemulsion is electrostatically stable as the zeta potential is below −30 mV and above +30 mV. The results of the zeta potential of blank and vitamin D_3_-loaded nanoemulsion are mentioned in Table 3. The values of zeta potential were found to be negative, ranging from −9.71 to −15.32 mV and −7.29 to −13.56 mV for blank and vitamin D_3_-loaded nanoemulsion, respectively. All the results of the zeta potential fall below ± 30 mV.

**Table 3:** Zeta potential of blank and vitamin D_3_-loaded nanoemulsion (mean ± SD *n* = 3).

	**Zeta potential (mV)**	
**Formulation code**	**Blank**	**Vitamin D_3_ loaded**
CA-1	−9.71 ± 0.48^c^	−7.29 ± 2.42^c^
CA-2	−11.74 ± 0.70^b^	−9.84 ± 0.59^b^
CA-3	−12.51 ± 0.37^ab^	−13.33 ± 4.50^a^
CA-4	−15.32 ± 0.03^a^	−13.56 ± 0.61^a^

Values are expressed as mean ± standard deviation (*n* = 3). Values in the same column within each parameter with different letters were significantly different from each other (*p* ≤ 0.05).

#### 3.3.4. pH and conductivity of nanoemulsion

The results of the pH and conductivity of the nanoemulsion are presented in Table 4. The pH of blank formulations was observed to range from 6.0 to 6.2, and the pH of vitamin D_3_-loaded formulations was found to range from 6.1 to 6.2, with slight variation in all formulations, which is considered a good candidate for oral administration (38). CA-3 treatment showed the best pH value of 6.2 for the oral administration of nanoemulsion. To determine the type of nanoemulsion, an electrical conductivity test was applied. Conductivity measurements of oil-in-water nanoemulsion have higher electrical conductivity values (10–100 μs/cm) (39). The electrical conductivity value before loading vitamin D_3_ ranged from 20 to 80 μsec/cm, and the vitamin D_3_-loaded nanoemulsion had an electrical conductivity ranging between 30 and 100 μsec/cm. Electrical conductivity values were found to decrease with the decrease in water content in the emulsions.

**Table 4:** pH and conductivity of blank and vitamin D_3_-loaded nanoemulsion (mean ± SD *n* = 3).

	**pH**		**Conductivity (**μ**S/cm)**	
**Formulation code**	**Blank**	**Vitamin D_3_ loaded**	**Blank**	**Vitamin D_3_-loaded**
CA-1	5.9 ± 0.04^a^	6.0 ± 0.04^a^	80 ± 4.71^a^	100 ± 0.0^a^
CA-2	6.0 ± 0.04^a^	6.1 ± 0.04^a^	50 ± 0.00^b^	80 ± 0.00^b^
CA-3	6.1 ± 0.04^a^	6.2 ± 0.08^a^	50 ± 0.94^b^	50 ± 0.00^c^
CA-4	6.2 ± 0.08^a^	6.3 ± 0.03^a^	20 ± 0.00^c^	30 ± 8.16^d^

Values are expressed as mean ± standard deviation (*n* = 3). Values in the same column within each parameter with different letters were significantly different from each other (*p* ≤ 0.05).

#### 3.3.5. Viscosity of nanoemulsion

Viscosity is another crucial consideration for the stability of nanoemulsions. The viscosity of nanoemulsion is a function of water, surfactant and co-surfactant concentration, and oil concentration. The results of viscosity are presented in Table 5. Viscosities of stable samples were found in the range of 0.54 mPa.s to 0.78 mPa.s of blank samples, and viscosities of vitamin D_3_-loaded nanoemulsions were observed to range from 0.61 mPa.s to 0.79 mPa.s. An increase in the viscosity of nanoemulsions might be due to an increase in oil content. The higher the oil content in the nanoemulsion, the higher will be the viscosity, and vice versa (40). As the oil content increased, it was discovered that CA-1 had a lower viscosity than CA-4 and that the viscosity of nanoemulsion was also rising. Because the blank sample had more oil than the vitamin D_3_-loaded formulation, the viscosities of the two were found to be different. By using dynamic light scattering (DLS) analysis, the nanoemulsions' particle size, polydispersity, and stability over time were examined. The polysorbate 20 and lecithin-based nanoemulsions that were investigated created systems with EVOO droplets that were stable for 37 days and measured 285 ± 5 nm in diameter and 0.202 ± 0.01 PdI. Calcium citrate and vitamin D3 were added to these durable nanoemulsions to create formulations that include both water- and oil-soluble micronutrients, as reported by Demisli et al. (41).

**Table 5:** Viscosity of blank and vitamin D_3_-loaded nanoemulsion (mean ± SD *n* = 3).

	**Viscosity (mPa.s)**	
**Formulation code**	**Blank**	**Vitamin D_3_ loaded**
CA-1	0.544 ± 0.02^c^	0.613 ± 0.17^c^
CA-2	0.658 ± 0.39^b^	0.665 ± 0.02^b^
CA-3	0.671 ± 0.01^b^	0.682 ± 0.41^b^
CA-4	0.789 ± 0.00^aa^	0.793 ± 0.01^a^

Values are expressed as mean ± standard deviation (*n* = 3). Values in the same column within each parameter with different letters were significantly different from each other (*p* ≤ 0.05).

### 3.4. Stability studies of nanoemulsion formulations

Based on visual observation, droplet size, zeta potential, pH, conductivity, and viscosity results, nanoemulsion formulation (CA-3) was selected for stability testing. There is no flocculation, and phase separations have appeared. All formulations were stable for more than 3 months as shown in Tables 9–11. The parameters including droplet size, polydispersity index, and zeta potential of formulation CA-3 (both blank and vitamin D_3_-loaded) were monitored at time zero; thereafter, 1, 2, and 3 months of storing at 25 ± 2 C/55% RH and 40 C/75% RH are shown in Tables 6–8. There is no difference in zeta potential, droplet size, or viscosity between ambient and accelerated storage conditions. Even after 3 months, there is no phase separation at room temperature or 40 C in nanoemulsion formulations. Morais et al. (42) conducted a related investigation as well. The purpose of this study was to assess the physicochemical properties of canola oil–water nanoemulsions, including zeta potential and droplet size values before and after stability testing. Owing to vitamin D's limited water solubility and degradation from exposure to the sun and high temperatures, fortifying foods and drinks with it are difficult, according to Mehmood and Ahmed (43). The results obtained imply that the nanoemulsion produced by the EPI and necessary HLB procedures is highly stable. The stated nanometer-sized droplets and strong zeta potential values can be supported by this shown in Tables 9–11. The tested parameters showed no discernible change, even though the emulsions were exposed to a variety of temperatures. Our study showed a slight decrease in viscosity with the elevation of temperature to 40 C, which was similar to the study described by Cheng et al. (44), whereas conductivity has not shown any changes during stability studies and PDI was also measured as < 1 (34). Similar results were reported by Zhang et al. (45). Vitamin D_3_ NEs showed greater stability under gastric digestion and prolonged release under gastrointestinal digestion as contrasted with unencapsulated (bare) vitamin D_3_. After 5 h of intestinal digestion, the total release of vitamin D_3_ reached 69%. Additionally, at 40–60°C, vitamin D_3_ NEs showed greater thermal stability than pure vitamin D3, and first-order kinetics was a good fit to characterize its thermal deterioration. Our research offers a fresh perspective on the use of vitamin D_3_ NEs stabilized by Span 80 and Tween 80 in the food sector. In systems stabilized by polysorbates, partial vitamin embedding that made the interface more fluid was also demonstrated. The vitamin-enriched nanoemulsions were then added to whole-fat milk, which remained resistant to particle development and gravitational dispersion for at least 10 days. Employing an EPR method, the antiradical capabilities of encapsulated cholecalciferol were examined and reported by Golfomitsou et al. (46).

**Table 6:** Stability data of droplet size at room temperature (RT) and 40°C for the nanoemulsion CA-3 formulation (both blank and vitamin D3-loaded). Mean ± SD, *N* = 3.

**Storage Conditions**	**Droplet size (d.nm)**				
	**Zero time**	**1 month**	**2 month**	**3 month**	**Mean**
RT (Blank)	66.48 ± 0.88	65.82 ± 0.61	66 ± 0.60	66.48 ± 0.02	66.20 ± 1.2^b^
RT (Vitamin D3-loaded)	185.5 ± 0.08	185.1 ± 0.08	186.1 ± 0.89	189.33 ± 2.89	186.51 ± 1.78^a^
40 C (Blank)	66.48 ± 0.88	66.48 ± 0.01	66.47 ± 0.01	66.49 ± 0.00	66.48 ± 2.43^b^
40 C (Vitamin D3-loaded)	185.5 ± 4.08	185.23 ± 0.32	187.83 ± 0.47	190.33 ± 1.64	187.22 ± 4.4^a^
Mean	125.99 ± 3.65^a^	125.6575 ± 1.13^a^	126.6 ± 3.56^a^	128.15 ± 2.23^a^	

Values are expressed as mean ± standard deviation (n = 3). Values in the same column within each parameter with different letters were significantly different from each other (p ≤ 0.05).

**Table 7:** Stability data of PDI at room temperature (RT) and 40°C for the nanoemulsion CA-3 formulation (both blank and vitamin D3-loaded).

**Storage conditions**	**PDI**				
	**Zero time**	**1 month**	**2 month**	**3 month**	**Mean**
RT (Blank)	0.51 ± 0.01	0.51 ± 0.02	0.50 ± 0.02	0.51 ± 0.01	0.51 ± 0.01^b^
RT (Vitamin D3-loaded)	0.93 ± 0.06	0.93 ± 0.01	0.93 ± 0.01	0.88 ± 0.03	0.92 ± 0.02^a^
40 C (Blank)	0.51 ± 0.01	0.51 ± 0.02	0.51 ± 0.01	0.51 ± 0.01	0.51 ± 0.01^b^
40 C (Vitamin D3-loaded)	0.93 ± 0.01	0.93 ± 0.01	0.90 ± 0.02	0.89 ± 0.01	0.91 ± 0.03^a^
Mean	0.720 ± 0.02^a^	0.720 ± 0.03^a^	0.710 ± 0.03^ab^	0.698 ± 0.02^b^	

Values are expressed as mean ± standard deviation (*n* = 3). Values in the same column within each parameter with different letters were significantly different from each other (*p* ≤ 0.05).

Mean ± SD, *N* = 3.

**Table 8:** Stability data of zeta potential at room temperature (RT) and 40°C for the nanoemulsion CA-3 formulation (both blank and vitamin D_3_-loaded).

**Storage conditions**	**Zeta Potential (mV)**				
	**Zero time**	**1 month**	**2 month**	**3 month**	
RT (Blank)	−15.32 ± 0.03	−15.36 ± 0.09	−15.41 ± 0.06	−15.36 ± 0.02	−15.36 ± 0.05^a^
RT (Vitamin D_3_-loaded)	−13.33 ± 4.50	−13.31 ± 0.01	−13.32 ± 0.02	−13.35 ± 0.00	−13.33 ± 0.03^b^
40°C (Blank)	−15.32 ± 0.03	−15.33 ± 0.01	−15.33 ± 0.01	−12.04 ± 4.73	−14.51 ± 0.01^a^
40°C (Vitamin) D_3−_loaded	−13.33 ± 4.50	−13.32 ± 0.01	−13.32 ± 0.02	−13.33 ± 0.01	−13.33 ± 0.03^b^
	−14.325 ± 0.02^a^	−14.333 ± 0.03^a^	−14.345 ± 0.04^a^	−13.521 ± 0.05^a^	

Values are expressed as mean ± standard deviation (*n* = 3). Values in the same column within each parameter with different letters were significantly different from each other (*p* ≤ 0.05).

Mean ± SD, *N* = 3.

**Table 9:** Stability data of pH at room temperature (RT) and 40 C for the nanoemulsion CA-3 formulation (both blank and vitamin D_3_-loaded).

**Storage conditions**	**pH**				
	**Zero time**	**1 month**	**2 month**	**3 month**	**Mean**
RT (Blank)	6.2 ± 0.08	6.1 ± 0.04	6.1 ± 0.12	6.1 ± 0.04	6.125 ± 0.04^a^
RT (Vitamin D_3_-loaded)	6.2 ± 0.08	6.2 ± 0.04	6.2 ± 0.04	6.1 ± 0.04	6.175 ± 0.04^a^
40°C (Blank)	6.2 ± 0.08	6.1 ± 0.04	6.2 ± 0.08	6.2 ± 0.09	6.175 ± 0.04^a^
40°C (Vitamin) D_3_-loaded	6.2 ± 0.08	6.2 ± 0.04	6.1 ± 0.04	6.0 ± 0.04	6.125 ± 0.04^a^
Mean	6.2 ± 0.02^a^	6.15 ± 0.01^a^	6.15 ± 0.03^a^	6.1 ± 0.01^a^	

Values are expressed as mean ± standard deviation (*n* = 3). Values in the same column within each parameter with different letters were significantly different from each other (*p* ≤ 0.05).

Mean ± SD, *N* = 3.

**Table 10:** Stability data of conductivity at room temperature (RT) and 40°C for the nanoemulsion CA-3 formulation (both blank and vitamin D_3_-loaded).

**Storage conditions**	**Conductivity (**μ**S/cm)**				
	**Zero time**	**1 month**	**2 month**	**3 month**	**Mean**
RT (Blank)	50 ± 0.94	50 ± 0.00	49 ± 0.47	52 ± 0.04	50.25 ± 0.01^a^
RT (Vitamin D_3_-loaded)	50 ± 0.00	51 ± 0.81	52 ± 0.81	51 ± 0.81	51 ± 0.02^a^
40°C (Blank)	50 ± 0.94	50 ± 0.47	51 ± 0.47	50 ± 0.00	50.25 ± 0.03^a^
40°C (Vitamin) D_3_-loaded	50 ± 0.00	51 ± 0.81	52 ± 0.04	51 ± 1.24	51 ± 0.01^a^
Mean	50 ± 0.02^a^	50.5 ± 0.01^a^	51 ± 0.03^a^	51 ± 0.02^a^	

Values are expressed as mean ± standard deviation (*n* = 3). Values in the same column within each parameter with different letters were significantly different from each other (*p* ≤ 0.05).

Mean ± SD, *N* = 3.

**Table 11:** Stability data of viscosity at room temperature (RT) and 40 C for the nanoemulsion CA-3 formulation (both blank and vitamin D3-loaded).

**Storage conditions**	**Viscosity (mPa.s)**				
	**Zero time**	**1 month**	**2 month**	**3 month**	**Mean**
RT (Blank)	0.67 ± 0.01	0.67 ± 0.02	0.65 ± 0.04	0.67 ± 0.06	0.67 ± 0.01^a^
RT (Vitamin D_3_-loaded)	0.68 ± 0.41	0.68 ± 0.00	0.68 ± 0.00	0.68 ± 0.00	0.68 ± 0.02^a^
40°C (Blank)	0.67 ± 0.01	0.68 ± 0.01	0.67 ± 0.00	0.66 ± 0.00	0.67 ± 0.03^a^
40°C (Vitamin) D_3_-loaded	0.68 ± 0.41	0.68 ± 0.00	0.67 ± 0.00	0.67 ± 0.00	0.68 ± 0.03^a^
Mean	0.675 ± 0.01^a^	0.677 ± 0.01^a^	0.667 ± 0.02^a^	0.671 ± 0.02^a^	

Mean ± SD, *N* = 3.

## 4. Conclusion

It was also observed that the primary size of nanoemulsion droplets depended on the type of S-Mix, S-Mix- level, and stirring conditions. It contains a droplet size of < 200 nm and could be developed by using a non-ionic surfactant and emulsifier (Tween 80 and Span 80) at a ratio of 1:1 with constant stirring during the titration process. By varying the composition of oil (5–20%), S-Mix (30–40%), and water (40–65%), 15 formulations were prepared. The outcomes delineated that the canola oil-based nanoemulsions proved efficient for the entrapment of vitamin D_3_, which can be used for the fortification of different food products to promote the delivery of this deficient micronutrient. Four nanoemulsion formulation combinations were finalized for the encapsulation of vitamin D_3_ through a phase diagram. Higher zeta potential, droplet size within an acceptable range, standard pH and conductivity, and stable viscosity were achieved in the CA-3 vitamin D_3_-encapsulated nanoemulsion. Thermal stability studies indicated that nanoemulsions stayed physically stable for 3 months at room temperature and also at an elevated temperature of 40 C. However, structural elucidation analysis such as FTIR should be performed in future studies to indicate bond formation.

## Data availability statement

The original contributions presented in the study are included in the article/supplementary material, further inquiries can be directed to the corresponding authors.

## Author contributions

AK: prepared original article. MA: supervision equal. AI: methodology equal. SH: methodology. MS: conceptulization equal. All authors contributed to the article and approved the submitted version.
